# Proceedings of 2011 Stillbirth Summit

**DOI:** 10.1186/1471-2393-15-S1-A2

**Published:** 2015-04-15

**Authors:** Edwin A Mitchell

**Affiliations:** 1 Department of Paediatrics, University of Auckland, Auckland, New Zealand

## 

The previous Stillbirth Summit presented by the Star Legacy Foundation and supported by various organisations was held in October 2011 in Minneapolis to discuss emerging ideas in the field of stillbirth research and management. In particular the focus was on the placenta, cord, infection and inflammation, reduced fetal movements and maternal sleep. Attendees were invited researchers, stillbirth advocates and parents. One of the strengths of the meeting was the robust debate amongst the researchers, alongside the energy and passion of the parents.

Unlike some scientific meetings which are talkfest, the Summit in 2011 had tangible outcomes. The first of which was that the majority of the researchers summarised their presentations, which were published in the BMC Pregnancy and Childbirth, which is an open access journal allowing anyone to access the content online [1]. Several important collaborations developed. Jane Warland and Ed Mitchell had independently developed conceptual models for the mechanism of stillbirths, which were adapted from the SIDS triple risk model. These authors collaborated in developing their ideas further and this has been recently published [2].

In 2011 Tomasina Stacey summarised the findings from The Auckland Stillbirth Study, a case-control study, which identified maternal non-left position on going to sleep was associated with a two fold increase risk of late stillbirths. The researchers urged caution and identified the need for robust, peer reviewed supporting evidence before recommending change or public health campaigns. Alex Heazell took up the challenge and with the support of the Auckland group has developed the Midland and North of England Stillbirth Study (MiNESS), which is funded by Action Medical Research and Cure Kids [3].

The most notable outcome was the development of the STARS Study, led by Louise O’Brien and Jane Warland, and supported by the Star Legacy Foundation. In essence this is an internet survey of women who had lost a baby in late pregnancy (28+ weeks gestation). There were two components, the first was women who had lost their baby more than 3 weeks prior to completing the survey. “The experiences of 1310 mothers of late stillbirths” was presented at the Stillbirth Summit 2014 by Jane Warland. The second component was a case-control study. The cases are mothers who had a late stillbirth less than 3 weeks prior to completing the interview. They are compared with women who had live ongoing pregnancies also at 28+ weeks gestation. The survey was extensive covering a wide range of issues. Recruitment has been difficult and the expected number of cases and controls are less than expected. Ed Mitchell presented interim findings from 132 cases and 283 controls. As these analyses were interim, the results are not presented here.
